# Evaluation of a New Smartphone Powered Low-cost Pulse Oximeter Device

**DOI:** 10.4314/ejhs.v32i4.22

**Published:** 2022-07

**Authors:** Hundessa Daba Nemomssa, Hakkins Raj

**Affiliations:** 1 School of Biomedical Engineering, Jimma Institute of Technology, Jimma University, Jimma, Oromia, Ethiopia

**Keywords:** COVID-19, Smartphone powered Pulse Oximeter, Performance evaluation, Accuracy, Low-cost

## Abstract

**Background:**

Measurement of blood oxygen saturation is a vital part of monitoring coronavirus 2019 (COVID-19) patients. Pulse oximetry is commonly used to measure blood oxygen saturation and pulse rate for appropriate clinical intervention. But the majority of direct-to-consumer grade pulse oximeters did not pass through in-vivo testing, which results in their accuracy being questionable. Besides this, the ongoing COVID-19 pandemic exposed the limitations of the device in resource limited areas since independent monitoring is needed for COVID-19 patients. The purpose of this study was to perform an in-vivo evaluation of a newly developed smartphone powered low-cost pulse oximeter.

**Methods:**

The new prototype of a smartphone powered pulse oximeter was evaluated against the standard pulse oximeter by taking measurements from fifteen healthy volunteers. The accuracy of measurement was evaluated by calculating the percentage error and standard deviation. A repeatability and reproducibility test were carried out using the ANOVA method.

**Results:**

The average accuracy for measuring spot oxygen saturation (SPO2) and pulse rate (PR) was 99.18% with a standard deviation of 0.57 and 98.78% with a standard deviation of 0.61, respectively, when compared with the standard pulse oximeter device. The repeatability and reproducibility of SPO2 measurements were 0.28 and 0.86, respectively, which is in the acceptable range.

**Conclusion:**

The new prototype of smartphone powered pulse oximeter demonstrated better performance compared to the existing low-cost fingertip pulse oximeters. The device could be used for independent monitoring of COVID-19 patients at health institutions and also for home care.

## Introduction

The ongoing COVID-19 pandemic has endangered the lives of people, resulting in more than 1.7 million deaths and over 79.2 million cases in 210 countries across the world (1). More than 80% of people infected with COVID-19 are asymptomatic or have moderate upper respiratory tract symptoms, which can lead to significant complications such as dyspnea, hypoxemia, acute respiratory distress syndrome (ARDS), shock, and even death (2,3). Oxygen is a cost-effective way of treatment for COVID-19 patients and it is a mandatory therapy due to the damage to the respiratory system resulting from the virus (4,5). However, oxygen therapy has to be given under proper monitoring of blood oxygen saturation in order to avoid hypoxia (6–10).

Pulse oximetry (PO) is a non-invasive method for the measurement of arterial oxygen saturation (SpO2) (11). PO is widely used as standard care in hospitals in developed countries (12,13), but its availability in developing country healthcare settings is limited (14), prompting the World Health Organization (WHO) to launch a global oximetry initiative to increase the availability of pulse oximeters (15). The challenge faced by health facilities in developing countries during the ongoing COVID-19 pandemic truly reflected the limitations of access to pulse oximeters, and a recent study on pulse oximeter availability in developing countries, specifically in Ethiopia, has reported a low availability of portable pulse oximeters in hospital settings (16). Despite the importance of accurately monitoring the blood oxygen saturation of patients receiving oxygen therapy (17–20), access to an independently used pulse oximeter has the potential to prevent the mortality of COVID-19 patients in low and middle-income countries. Traditional pulse oximeter devices, on the other hand, are expensive, bulky, and unsuitable for use in low-resource settings (21,22).

Several portable and advanced pulse oximeters have recently been developed and their performance evaluated. For instance, Peterson et al. (23) developed and evaluated a low-cost smartphone pulse oximeter and obtained root mean square accuracy in the range of International Organization for Standardization (ISO) 4% for SPO2 measurement without giving emphasis to pulse rate and specifying the cost of the device. Huang et al. (24) developed a ring-type pulse oximeter with a multi-detector and obtained an SPO2 correlation of 98.26% compared with a commercially available pulse oximeter without considering the measurement of pulse rate and device accessibility in resource limited areas. In the same manner, Lin et al. (25) developed a wearable and wireless finger base-type pulse oximeter, focusing only on the measurement of oxygen saturation. Smartphone app pulse oximeters were also developed with a wide range of accuracy, but the accessibility and reliability of these developments were limited (26). On the other hand, Shrading et al. (27) compared the SPO2 measurement accuracy of three consumer grade pulse oximeters by comparing them with bedside pulse oximeters and obtained clinically significant accuracy even though the test characteristics were not perfect and the pulse rate was not considered.

In this study, the performance of a new prototype of a smartphone powered, low-cost portable pulse oximeter developed for independent use by COVID-19 patients was evaluated by comparing it with the standard device and taking measurements from healthy volunteers. During performance evaluation, SPO2 and pulse rate measurement accuracy were considered under low perfusion index.

## Materials and Methods

**Figure 1:**
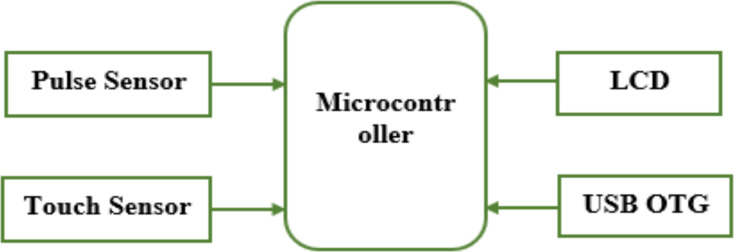
Block Diagram of smartphone powered low-cost pulse oximeter device

**System design**: The smartphone powered low-cost pulse oximeter was developed based on the block diagram shown in Figure 1.

**Figure 2:**
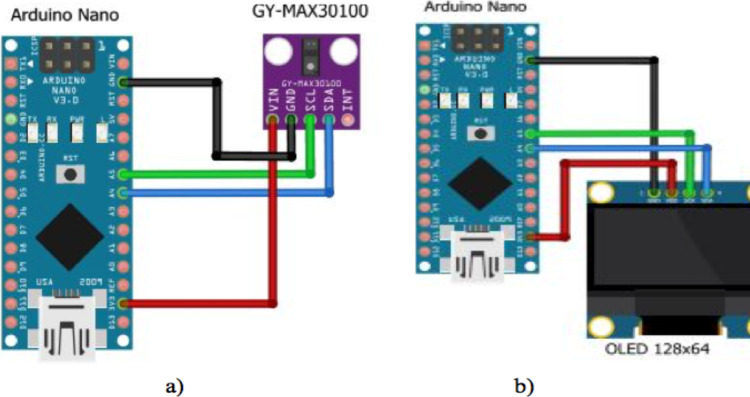
Connection between a) Pulse sensor and Arduino Nano b) OLED Display and Arduino nano

**Figure 3:**
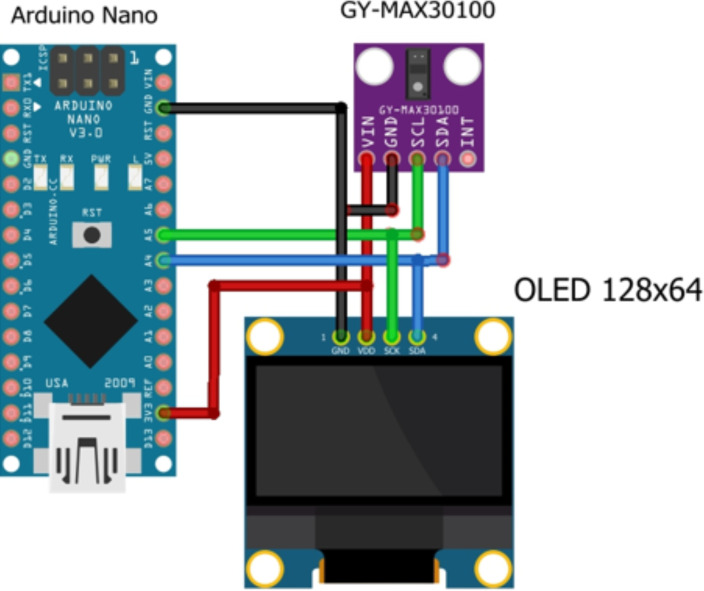
Wiring diagram of Smartphone powered low-cost pulse oximeter device.

The pulse sensor (GY-MAX30100) measures the SPO2 and pulse rate and sends the data to the Arduino nano microcontroller through the I2C protocol. I2C is a popular serial communication technology that is used in embedded devices. A touch sensor is used to allow the user to put their finger appropriately on the pulse sensor. An OLED 128x64 display having a 1.8-inch screen size was used to display the measurement result for the user. A Universal serial port on-the-Go (USB OTG) was used to connect the pulse oximeter with the smart phone through a USB cable to power the device. The wiring diagram showing the connection between the pulse sensor and the Arduino nano microcontroller and between the OLED display and the Arduino microcontroller is shown in Figure 2, while the overall wiring diagram used for development of the device is shown in Figure 3.

**Figure 4:**
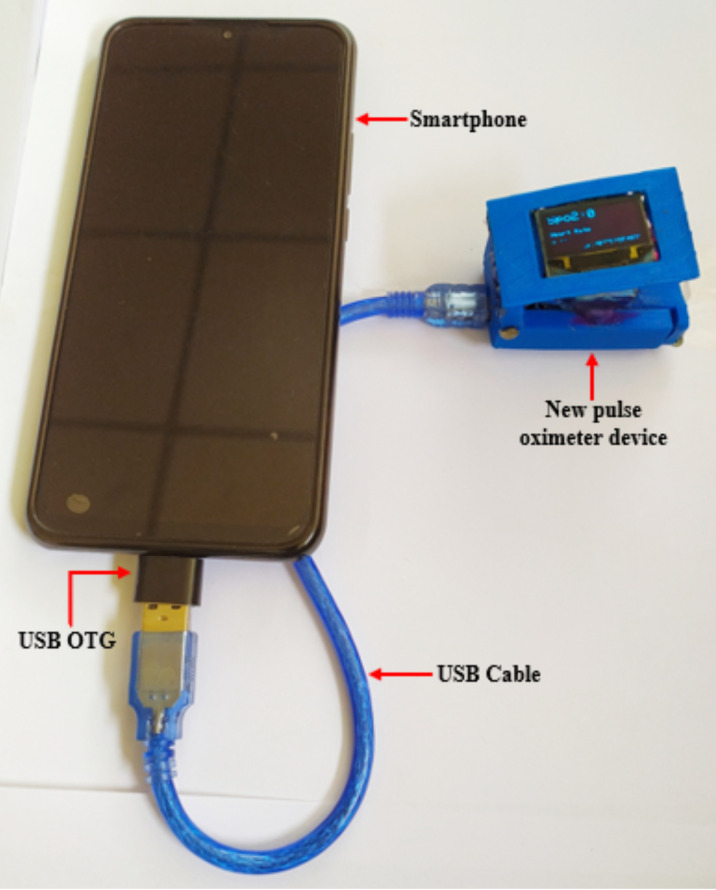
Prototype of a new smartphone powered low-cost pulse oximeter

**Software and hardware development**: The Arduino integrated development environment (IDE) was used to write the program code that governs the operation of the device. The device cover was designed using Automated Computer Aided Design (AutoCAD) software and was printed using a 3D printer. The electrical components were connected using jumper wires based on the wiring diagram shown in Figure 3 and finally assembled in the device cover. Figure 4 shows the prototype of a new smartphone powered low-cost pulse oximeter.

**Performance evaluation**: The device's performance was evaluated by taking the measurement of fifteen (15) healthy volunteers and comparing the results with the standard pulse oximeter device. The percent error between the standard device and our device was calculated using equations 1 and 2 from (28).


(1) $$\%\;\mathrm{ErrorSPO}2=\left(\frac{\mathrm{\Delta SPO}2(\%)}{\mathrm{SPO}2\;\mathrm{measured}\;\mathrm{by}\text{ standard }\mathrm{device}\;(\%)}\right)x\;100\%$$

(2) $$\%\;\mathrm{ErrorPR}=\left(\frac{\mathrm{\Delta PR}(\mathrm{bpm})}{\mathrm{PR}\;\mathrm{measured}\;\mathrm{by}\text{ standard }\mathrm{device}\;(\mathrm{bpm})}\right)x\;100\%$$

## Results

The performance of the new pulse oximeter was evaluated with tests for oxygen saturation and pulse rate against the standard handheld pulse oximeter. The results show a strong correlation between SPO2 and pulse rate values obtained by the new pulse oximeter device and a standard pulse oximeter device. The minimum percentage error for the measurement of SPO2 was 0% and the maximum percentage error obtained was 2.04% compared with the standard device. On the other hand, the minimum percentage error of 0% and the maximum percentage error of 1.85% were obtained for the measurement of pulse rate. The root mean square accuracy (ARMS) for the measurement of SPO2 by the new pulse oximeter is 1.02 while it is 1.32 for pulse rate measurement. The details of the performance evaluation against the standard device are summarized in Table 1.

**Table 1:** Performance Evaluation of smartphone powered pulse oximeter with standard device

Volunteer Number	SPO2 Measurement			Pulse rate measurement		

	Using standard Pulse oximeter	Using our Device	% Error	Using standard Pulse oximeter	Using our Device	% Error
1	96	97	1.04	74	75	1.35
2	96	96	0.00	81	82	1.23
3	97	97	0.00	65	66	1.54
4	97	97	0.00	82	81	1.22
5	98	96	2.04	71	72	1.41
6	97	96	1.03	54	55	1.85
7	97	96	1.03	63	63	0.00
8	98	97	1.02	76	77	1.32
9	96	96	0.00	73	74	1.37
10	99	97	2.02	68	69	1.47
11	95	96	1.05	74	74	0.00
12	97	98	1.03	71	70	1.41
13	98	97	1.02	76	77	1.32
14	97	97	0.00	69	68	1.45
15	96	95	1.04	73	74	1.37
**Average % Error**			**0.82**			**1.22**

The average accuracy for measuring SPO2 and pulse rate was 99.18% with a standard deviation of 0.57 and 98.78% with a standard deviation of 0.61, respectively. In addition to the accuracy test, the device's repeatability and reproducibility were evaluated using the ANOVA method. Accordingly, the repeatability and reproducibility of SPO2 measurements were 0.28 and 0.86, respectively, which is within the acceptable range.

## Discussion

Pulse oximeters are very important for monitoring COVID-19 patients at all levels of health care institutions because COVID-19 causes silent hypoxia without shortness of breath that leads to lung damage, further difficulties, and mortality (29–31). Early detection of this silent hypoxia in COVID-19 patients is therefore critical in preventing morbidity and death (32,33).

Despite the widespread availability of direct-to-consumer grade pulse oximeters on the market, the majority of them did not go through rigorous in vivo testing, and thus their accuracy is unknown (34). According to the study done by Harskamp et al., only five out of ten commercially available pulse oximeters met the accuracy standards set by Food and Drug Administration (FDA) and International Organization for Standardization (ISO) standards, while none of them met the root mean square accuracy requirement of ≤ 3% (34).

We conducted an in-vivo evaluation of a novel smartphone-powered pulse oximeter based on a standard handheld pulse oximeter and found that our device's oxygen saturation and pulse rate output had great correlation and a low percentage error. The SPO2 correlation obtained in this study is better than the SPO2 correlation obtained by previous studies, such as the 98.26% SPO2 correlation obtained by Huang et al. (24) and a better root mean square accuracy was obtained compared with the six commercially available low-cost fingertip pulse oximeters costing less than $50 as shown in Table 2. Despite this, our new device has a cost advantage since these commercially available pulse oximeters have an additional cost for battery while ours is powered by a smartphone (35).

**Table 2:** Comparison of the smartphone powered pulse oximeter with commercially available fingertip pulse oximeters

Authors	Finger pulse oximeter Brand	A_rms_ (%) for oxygen saturation 90%–100%
Lipnick et al. (35)	Starhealth SH-A3	1.36
	Jumper FPD-500A	1.25
	Atlantean SB100 II	1.78
	Contec CMS50DL	1.98
	Beijing Choice C20	1.21
	Beijing Choice MD300C23	2.17
**The current study**	**Smartphone powered** **low-cost pulse oximeter**	**1.06**

The results from this study demonstrated good performance under low peripheral perfusion situations, which is comparable to existing reflective type pulse oximeters (36,37). With the current evaluation, the new smartphone powered low-cost pulse oximeter meets the required standard for measurement of oxygen saturation and pulse rate (38). The device could be used for independent monitoring of COVID-19 patients at health institutions and also for home care. In the future, factors that affect the reliability of pulse oximeters, such as the effects of moisture in terms of sweat, temperature, tremors due to chillness, external light sources, external vibrations, anemia, nail polish, and dark skin pigmentation (34) should be incorporated into the performance evaluation.
